# Mapping and Identifying a Candidate Gene *Plr4*, a Recessive Gene Regulating Purple Leaf in Rice, by Using Bulked Segregant and Transcriptome Analysis with Next-Generation Sequencing

**DOI:** 10.3390/ijms20184335

**Published:** 2019-09-04

**Authors:** Ju Gao, Gaoxing Dai, Weiyong Zhou, Haifu Liang, Juan Huang, Dongjin Qing, Weiwei Chen, Hao Wu, Xinghai Yang, Danting Li, Lijun Gao, Guofu Deng

**Affiliations:** 1 Guangxi Crop Genetic Improvement and Biotechnology Laboratory, Guangxi Academy of Agricultural Sciences, Nanning 530007, China; 2 Rice Research Institute, Guangxi Academy of Agricultural Sciences, Nanning 530007, China

**Keywords:** *Oryza sativa*, purple leaf, candidate gene, BSA-Seq, RNA-Seq

## Abstract

The anthocyanin biosynthesis of rice is a major concern due to the potential nutritional value. Purple appears in various organs and tissues of rice such as pericarp, flower organs, leaves, leaf sheaths, internodes, ligules, apex, and stigma. At present, there are many studies on the color of rice pericarp, but the gene and mechanism of other organs such as leaves are still unclear, and the gene regulatory network of specific organ coloring has not been systematically understood. In this study, genetic analysis demonstrated that the purple leaf traits of rice were regulated by a recessive gene. The green leaf cultivar Y58S and purple leaf cultivar XianHongB were used to construct the mapping population. A set of near isogenicline (NIL) (BC_3_F_1_) was bred via crossing and back-crossing. The generations of BC_3_F_2_ appeared to separate four phenotypes, pl1, pl2, pl3, and pl4, due to the occurrence of a purple color in different organs. We constructed three bulked segregant analysis (BSA) pools (pl1–pl2, pl1–pl3, and pl1–pl4) by using the separated generations of BC_3_F_5_ and mapped the purple leaf gene *plr4* to the vicinity of 27.9–31.1 Mb on chromosome 4. Subsequently, transcriptome sequencing (RNA-Seq) for pl3 and pl2 was used to analyze the differentially expressed genes in the localization interval, where 12 unigenes exhibited differential expression in which two genes (*Os04g0577800*, *Os04g0616400*) were downregulated. The two downregulated genes (*Os04g0577800* and *Os04g0616400*) are possible candidate genes because of the recessive genetic characteristics of the purple leaf genes. These results will facilitate the cloning of *plr4* and illustrate the molecular mechanisms of the anthocyanin synthesis pathway.

## 1. Introduction

Anthocyanin is a strong antioxidant, which brings various colors to plant organs and exerts obvious effects on human health. The anthocyanin biosynthetic pathway is a branch of the plant flavonoid pathway, and its biosynthetic pathway has been relatively clear in the study of model plants such as *Arabidopsis thaliana*, maize, and petunia, where many key genes have been cloned. Anthocyanin biosynthesis is jointly controlled by two types of genes: structural genes including *CHS*, *CHI*, *F3H*, *F3′H*, *F3′5′H*, dihydroflavonol-4-reductase(*DFR*), *ANS*, and *3GT* [1,2,3,4,5,6,7], which encode various enzymes required for anthocyanin biosynthesis pathways [8]; and regulatory genes, which encode transcription factors that regulate the spatio-temporal expression of structural genes including the R2R3-MYB protein, MYC family of basic helix–loop–helix (bHLH) proteins, and WD40 protein gene family. The regulatory process may be completed by a complex comprising a transcription factor containing a R2R3-Myb DNA binding domain, a transcription factor containing a bHLH domain, and a protein containing WD repeats. These two types of genes work together to control the distribution of anthocyanins in plants organs [9,10]. The *R* gene family including *R*, *B*, *Lc*, and *Sn* genes regulate the organ-specific pigmentation patterns [11].

Rice is one of the most important food crops for humans, and anthocyanin biosynthesis has been widely studied in rice. Through homologous gene cloning, the genes of *CHS*, *CHI*, *ANS*, and *DFR* have been identified in rice [12,13,14]. Some anthocyanin transcription factors have been reported such as *OsC1*, *Ra*, *Rb*, *Rc*, *Rd*, *OsB1*, and *OsB2*. The *OsC1* gene of rice is highly homologous to the *C1* gene on chromosome 9 of maize, which is located on the short arm of chromosome 6 [15] and encodes the transcription factor of the R2R3-Myb family; this family of transcription factors plays a key role in the biosynthesis of anthocyanin in rice [12,16,17,18,19]. The genes of *Ra*, *Rb*, *Rc*, and *Rd* belong to the *R/B* gene family. *Ra* is the homologous gene of the maize *R/B* gene that is located on chromosome 4 of rice, encodes a transcription factor of the bHLH domain, is composed of two genes *Ra1* and *Ra2*, and plays an important role in the anthocyanin biosynthesis pathway. It is an important gene for black pericarpin rice [20]. The *Rb* gene, which is located on chromosome 1 of rice, can induce pigment accumulation in maize suspension cells [21]. The *Rc* and *Rd* genes, which encode bHLH protein, are positive regulators of proanthocyanidin synthesis, which is related to the color of the testa [22,23]. The *Rc* gene, which is located on chromosome 7 of rice, encodes the bHLH class transcription factor [22]. The *Rd* gene is homologous to the *A* gene of maize and is located on chromosome 1, which encodes *DFR* [23].

The accumulation of flavonoids, organs, and tissues in rice (*Oryza sativa*) can be purple, brown, or red in color. The potential nutritional value of colored pericarp has attracted attention, with geneticists and breeders having conducted a lot of research on this area. However, its location distribution mechanism in various tissues and organs has not been clearly studied. The accumulated anthocyanin of purple pericarp (or black rice) can be controlled by three alleles loci for *Kala1*, *Kala3*, and *Kala4* (*OsB2*) [24,25]. *Kala4*, a transcriptional regulator of *myc* type in rice, is located at the same gene locus as *OsB2*. It has been reported that *OsB2* is related to the accumulation of pigments in rice leaves [26]. Oikawa et al. found that the promoter structure of *OsB2* (*Os04g0557500*) gene was rearranged due to the insertion of a retrotransposon, which led to the ectopic expression of *OsB2* and the production of black rice [27]. The red pericarp is produced by the accumulation of proanthocyanidins. The synthesis of proanthocyanidins is regulated by the interaction of *Rc* (bHLH) and *Rd* (DFR) genes. The production of brown pericarp is involved only in the *Rc* gene, but not in the *Rd* gene [22,23]. The *C–S–A* gene model was proposed for rice hull pigmentation. *C1* (*Os06g0205100*, *OsC1*) is a color-producing gene that acts as a switch in controlling color production and produces a brown color when working alone, but produces a purple color when used in combination with *A1* (*Os01g0633500*, *DFR*). In addition, purple and brown hulls require a functional *S1* (*Os04g0557500*); otherwise, these two colors will only occur in the apiculus [28]. Transcriptome and proteome profiling of red, black, and white rice revealed 32 genes involved in the flavonoid biosynthesis pathway on the basis of differential enrichment analysis in which only CHI, F3H, ANS, and FLS were detected by isobaric tags for relative and absolute quantification analyses [29].

A purple color occurs in organs and tissues such as pericarp, floral organs, leaves, leaf sheaths, internodes, ligules, apiculus, and stigma. Up to now, there have been many studies on the pericarp color of rice, but the pattern and gene of other organ coloration are still unclear, and the gene regulatory network of specific organ coloration has not been systematically understood. Moreover, the *pl^w^* locus contains two adjacent genes, *OsB1* and *OsB2*, which are responsible for leaf coloration, but their functions at the molecular level have not been clarified [26]. The *pl^w^* gene is located on chromosome 4 of rice and is a regulatory factor controlling the synthesis of anthocyanidin in rice leaf tissue. The *pl^w^* gene not only stains all aerial tissues, except for the internodes, but also displays purple pericarp [26]. The *OsB1* and *Ra1* genes are the same gene loci [20]. The *OsB2* gene is a *myc* transcriptional regulator in rice that is involved in pigment accumulation in rice leaves. In black pericarp rice, the promoter structure of the *OsB2* gene is rearranged due to the insertion of a retrotransposon, resulting in the ectopic expression of *OsB2* and the production of black pericarp rice [27].

Purple leaf trait is a special leaf color marker trait with a stable expression and easy identification during the whole growth period. In addition to the molecular mechanism of anthocyanin regulation, it can be used in hybrid rice breeding such as two-line hybrid rice. Considering its own seed quality or the influence of weather, the two-line sterile line of rice often has 3–5% self-crossing hybrid seeds, which causes a loss of 500–600 million kg of rice annually in China. When the purple leaf character is introduced into the two-line sterile line, the true hybrid and self-fertile seeds can be identified based on leaf color, which can effectively and conveniently solve the problem of false hybrid identification in hybrid rice seed production.

In this study, genetic analysis demonstrated that the purple leaf traits of rice were regulated by recessive genes. Through bulked segregant analysis (BSA) with next-generation sequencing (BSA-Seq) and transcriptome sequencing (RNA-Seq) strategies, we identified *plr4*, which is a new gene that regulates purple leaf in rice.

## 2. Results

### 2.1. Statistical Analysis of Phenotypes and Genetics of Rice’s Purple Leaf Trait

**Figure 1:**
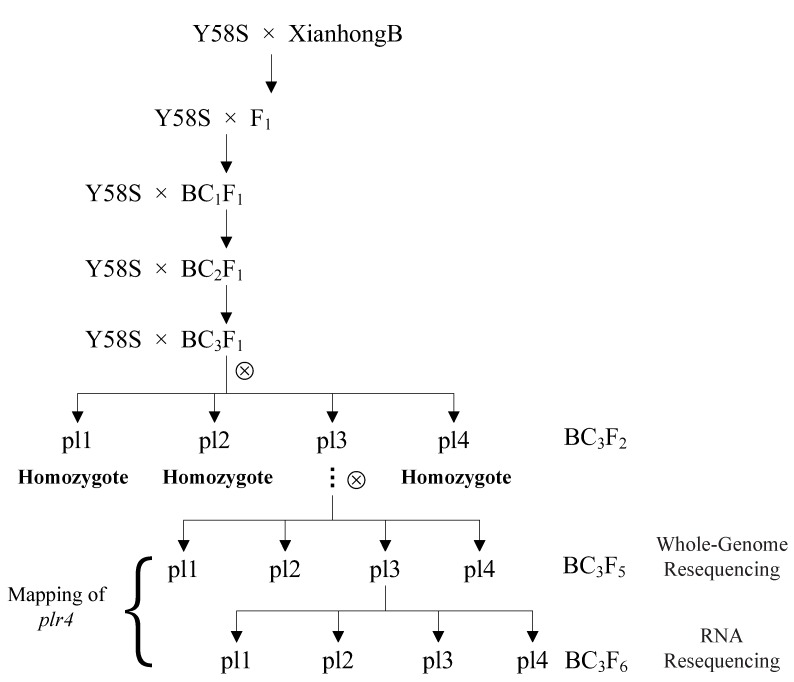
Construction of mapping population.

**Figure 2:**
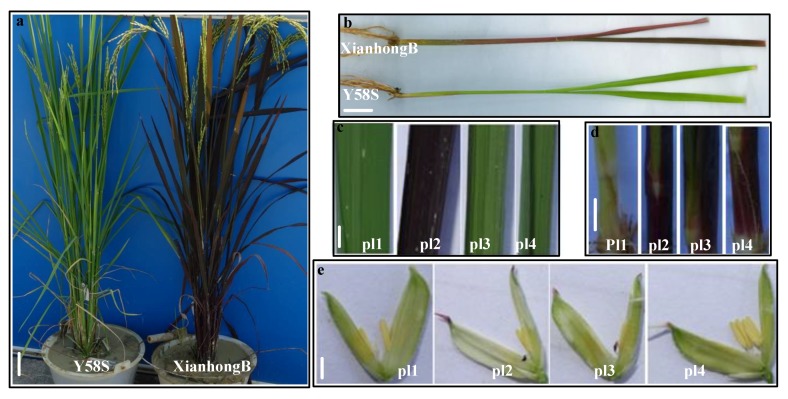
Phenotypic characteristics of purple leaf for different tissues and growth stages of rice. (**a**) heading stage, (**b**) seedling stage, (**c**) leaf, (**d**) leaf sheath, and (**e**) flower organ. The bar sizes are (**a**) 5 cm, (**b**), (**c**), (**d**), and (**e**) 1 cm.

The rice variety XianhongB, a fertility restorer line with purple coloration in the leaves, leaf sheaths, leaf rings, stems, and lemma, was selected as the donor parent. The rice variety Y58S, a male sterile line that appears as a whole green plant and with white stigma, was selected as the recipient parent. The plants of the F1 hybrid by crossing XianhongB with Y58S demonstrated green leaves and purple coloration in the leaf sheaths, leaf rings, stems, and lemma, indicating that the purple leaf of this rice variant was controlled by the recessive gene. A set of near isogenic lines (BC3F1) was bred via crossing and back-crossing with XianhongB and Y58S. The generations of BC_3_F_2_ were separated into four phenotypes: pl1, pl2, pl3, and pl4. pl1 was a whole green plant and with white stigma, the same as Y58S. pl2 exhibited purple coloration in the leaves, leaf sheaths, leaf margin, lemma tip, and stigma with the same traits as XianhongB. pl3 showed green leaves with purple coloration in the leaf sheaths, leaf margin, lemma tip, and stigma. pl4 presented green leaves and white stigma with purple coloration in the leaf sheaths, leaf margin, and lemma tip. The offspring of pl1, pl2, and pl4 were not separated in phenotype. The phenotype of the offspring of pl3 was separated into pl1, pl2, pl3, and pl4. Hence, the separated generations of BC_3_F_5_ and BC_3_F_6_ of pl3 were selected to map the purple leaf gene *plr4* (Figure 1 and Figure 2).

### 2.2. Identification of Candidate Intervals via Whole-Genome Resequencing Analysis

**Figure 3:**
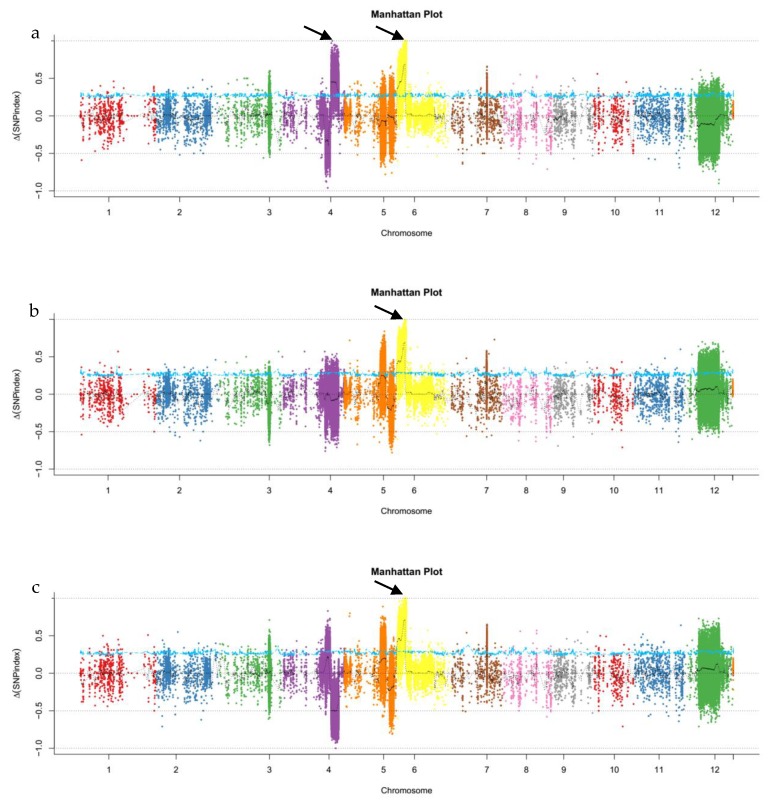
Two generations of the delta_All-index distributed on chromosomes. (**a**) pl1–pl2 pool, (**b**) pl1–pl3 pool, and (**c**) pl1–pl4 pool. The black arrow points to the BSA location range.

A total of 90.23 G of raw data were obtained and filtered into 89.94 G of clean data. We analyzed the quantity and quality of the data and found that Q20 ≥ 96.78% and Q30 ≥ 94.60%. GC content of the six samples’ clean data ranged from 42.40% to 44.30%. The average reading depth of six samples ranged from 31.04× to 53.58×, and the 95% confidence interval of SNP index for each reading depth was obtained. (Tables S1 and S2). The clean data were aligned to the reference genome. More than 2.55 million, 2.55 million, and 2.54 million SNPs and 0.47 million, 0.47 million, and 0.47 million indels were identified between the pl2 and pl1 pools, the pl3 and pl1 pools, and the pl4 and pl1 pools, respectively (Tables S3 and S4). The △All-index (△SNP-index and △Indel-index) was calculated, and a chart was plotted from the △All-index of every two sample pools (Figure 3). Two different intervals for the candidate gene that exceeded the threshold value were identified on the 27.9–31.1 MB of chromosome 4 and on the 1.9–5.4 MB of chromosome 6 via BSA analysis between the pl2 and pl1 pools. Through two BSA analyses between the pl3 and pl1 pools and between the pl4 and pl1 pools, the intervals for the candidate gene of the pl3 and pl4 phenotypes that exceeded the threshold value were identified on the 1.2–5.4 Mb and 0.9–5.4 Mb of chromosome 6, respectively. The findings were in accordance with the intervals in chromosome 6 of the BSA analysis between the pl2 and pl1 pools. These results suggest that the candidate genes of purple leaf rice are located in the 3.2 Mb region of chromosome 4 from 27.9 Mb to 31.1 Mb (Figure 3 and Figure S1).

### 2.3. Identification of Expressed Genes in a Single Candidate Interval via Transcriptome Sequencing

A total of 57,867,934–62,400,200 raw 150 bp paired-end reads were obtained from three green leaf samples (pl3) and three purple leaf samples (pl2) via RNA sequencing. After trimming the raw data, an average of 8.94 and 8.56 G clean bases remained from the pl2 and pl3 samples, respectively. The quantity and quality (Q20 ≥ 94.70% and Q30 ≥ 92.11%) of the data were analyzed. The GC content ranged from 57.75% to 60.38% (Table S5).

After mapping to the reference genome, a total of 92,028 unigenes were compared, and 19,970 and 19,733 genes were identified in the pl2 and pl3 plants, respectively (Table S6 and Figure S2). A total of 181 unigenes exhibited differential expression levels between pl2 and pl3 plants at a false discovery rate (FDR) ≤0.05 and |log2FC| ≥ 1 including 154 upregulated and 27 downregulated genes (Table S7). The most enriched gene ontology (GO) terms focused on metabolic process, catalytic activity, and transferase activity (Figures S3 and S4).

**Figure 4:**
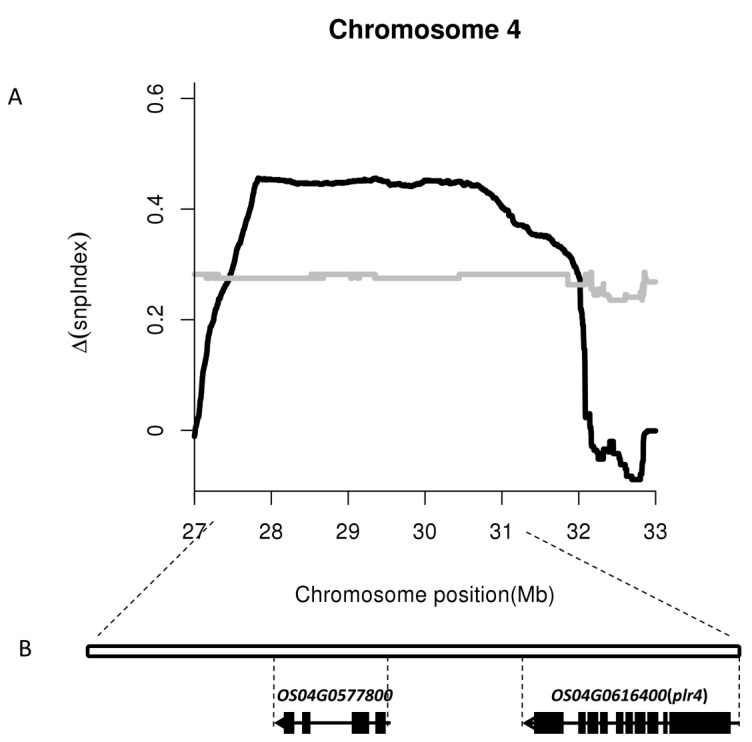
Identification and validation of the plr4 gene on chromosome 4. (**A**) Distribution of △(SNP-index) on chromosome 4; and (**B**) location of plr4 via whole-genome sequencing and RNA-Seq.

With the 3.2 Mb region of chromosome 4 (27.9–31.1 Mb), 327 unigenes were identified; 230 of the unigenes were expressed, and 12 unigenes exhibited differential expression in which only two genes (*Os04g0577800*, *Os04g0616400*) were downregulated. The two unigenes were located near the 27.9–31.1 Mb interval in the BSA analysis (Table S7 and Figure 4). Given the recessive genetic characteristics of the purple leaf genes, these two downregulated genes (*Os04g0577800* and *Os04g0616400*) are possible candidate genes.

In addition, 19 DEGs demonstrated annotation functions related to the anthocyanin pathway (Table 1). Among these genes, six genes (*OS02G0207100*, *OS04G0305700*, *OS04G0525100*, *OS04G0525200*, *OS06G0187500*, and *OS12G0561900*) encode UDP-GLYCOSYLTRANSFERASE, four genes (*OS01G0735300*, *OS06G0192100*, *OS07G0503500*, and *OS11G0461200)* encode anthocyanidin 3-O-glucosyltransferase, two genes (*OS04G0557800* and *OS11G0258700*) encode anthocyanin regulatory Lc protein, and seven genes (*OS01G0372500*, *OS01G0633500*, *OS04G0557500*, *OS04G0630300*, *OS10G0320100*, *OS11G0530600*, and *OS12G0270900*) encode leucoanthocyanidin dioxygenase, DFR, anthocyanin regulatory R-S protein, anthocyanidin reductase, flavonoid 3′-hydroxylase, chalcone synthase 1, and flavonol 3-sulfotransferase, respectively. Except that *OS02G0207100*, *OS04G0630300*, and *OS12G0561900* were downregulated, the other 16 genes were upregulated in purple leaf rice.

### 2.4. Verification of DEGs via qRT-PCR

**Figure 5:**
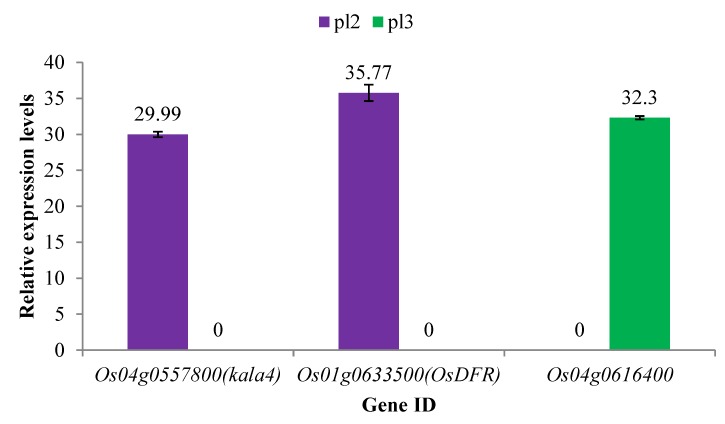
qRT-PCR to verify the DEGs via RNA-Seq. The abscissa is the gene accession, and the ordinate is the relative expression level of the gene.

To confirm that the genes identified within the 27.9–31.1 MB interval from RNA sequencing were differentially expressed, we selected one candidate gene (*Os04g0616400*) and two DEGs (*Os04g0557800* and *Os01g0633500*) related to anthocyanin, and qRT-PCR analysis was used to verify the expression of DEGs in seedlings (Table S8). The results showed that these genes exhibited the same expression trend as RNA-Seq data (Figure 5). It can be seen that the reliability of the transcriptome analysis is sustained.

### 2.5. Kyoto Encyclopedia of Genes and Genomes (KEGG) Enrichment of DEGs in the Anthocyanin Biosynthesis Pathway

KEGG pathway enrichment revealed six DEGs in the anthocyanin biosynthesis pathway; five DEGs (*OS11G0530600*, *OS04G0662600*, *OS10G0320100*, *OS01G0633500*, and *OS01G0372500*) were upregulated, and only one DEG (*OS04G0630300*) was downregulated (Figures S5 and S6). The five upregulated genes were distributed throughout the anthocyanin synthesis pathway. The downregulated gene was the anthocyanidin reductase gene, which was located in the downstream of the proanthocyanidin biosynthesis pathway and functions independent of anthocyanin synthesis regulation.

## 3. Discussion

In the past few decades, molecular markers have played an important role in target gene location and selective breeding. However, the effect of gene mapping in different populations varies, and linkage mapping is difficult to carry out on some groups [30]. The purple leaf characters are regulated by multiple genes [20,26,27], and several related genes are located in the adjacent regions of chromosome 4 including the *plr4* gene. We constructed the NILs, which could not be successfully mapped in the genome interval by molecular markers (data not shown).

The development of NGS technology can compensate for these deficiencies and promote the development of plant molecular breeding [31,32,33,34]. BSA mixed population separation analysis is often used in whole-genome resequencing, and two groups with extreme traits are mainly sequenced by mixed pool. The significant difference between the allele frequency (AF) of the polymorphic loci (SNP) of the two populations was compared to achieve the target of location-related genes and loci. RNA-seq can almost obtain the expression of all genes (including low abundance genes), evaluate the expression changes of various genes, and determine new genes and SNP loci [29]. In this study, NGS and RNA-seq were used to successfully locate a recessive purple leaf regulatory gene and screen its candidate genes.

Although several genes related to anthocyanin synthesis and regulation have been found, the molecular basis for the synthesis and regulation of anthocyanins in rice leaf tissue remains unclear. Given the interaction of polygenes and their genetic specificity, the recessive regulatory genes of the purple leaf of rice have been puzzled by the construction of the mapping population and gene cloning. At present, only the *OsB2* (*Os04g0557500*) gene on chromosome 4 has been found to regulate the purple leaf in rice. Functional verification of *OsB2* has only been reported for the ectopic expression of pericarp due to the rearrangement of its promoters [27]. These results have not clearly explained the molecular regulation mechanism of purple leaf in rice.

**Figure 6:**
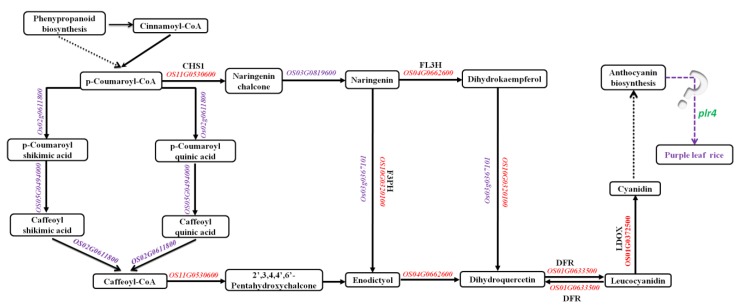
The predicted molecular mechanism of purple leaf formation in rice. Purple is the expression gene, red is the upregulation expression gene, and green is the downregulation expression gene.

In this study, we identified the recessive regulatory gene *plr4* of the purple leaf in rice, which was located near the 27.9–31.1 Mb interval of chromosome 4 (Figure 3). Subsequently, we analyzed the DEGs via transcriptome sequencing, where 19 DEGs demonstrated annotation functions related to the anthocyanin pathway (Table 1) including six UDP-glucosyltransferase genes, four anthocyanidin 3-O-glucosyltransferase genes, two Lc protein genes, and one each of leucoanthocyanidin dioxygenase, DFR, anthocyanin regulatory R-S protein, anthocyanidin reductase, flavonoid 3′-hydroxylase, chalcone synthase 1, and flavonol 3-sulfotransferase. Except that *OS02G0207100*, *OS04G0630300*, and *OS12G0561900* were downregulated, the other 16 genes were upregulated in purple leaf rice. The genes *OsB1* (*Os04g0557800, Kala4*) and *OsB2* (*Os04g0557500, Kala4*) were upregulated in purple leaf rice, which further confirmed its association with anthocyanin [20,25,27]. On chromosome 4, only six genes were remarkably downregulated in purple leaf rice in which only two genes (*Os04g0577800* and *Os04g0616400*) were located near the 27.9–31.1 Mb interval in the BSA analysis (Figure 4). *Os04g0577800* (chr4: 29,124,636–29,126,983 bp, complement) is a fatty acid output protein gene in the chloroplast, which is a gene with relatively low expression in the purple leaf rice that we detected. *Os04g0616400* (chr4: 31,285,362–31,298,409 bp, complement) has been annotated to a gene associated with serine protein kinase in *Arabidopsis thaliana* and demonstrates transferase activity. The read count of the gene expression was 0 in the samples of purple leaf rice we detected, but it is highly expressed in green leaf rice. Therefore, *Os04g0616400* is a candidate gene for *plr4*, whose function in rice has not been confirmed, and its molecular basis remains unclear. KEGG analysis revealed that the anthocyanin biosynthesis pathway in rice purple leaves was probably co-regulated by *plr4* and several anthocyanin-related genes *OS11G0530600* (chalcone synthase), *OS04G0662600* (flavanone 3-dioxygenase), *OS10G0320100* (flavonoid 3′-monooxygenase), *OS01G0633500* (DFR), and *OS01G0372500* (leucoanthocyanidin dioxygenase). We speculate that the *plr4* gene is the downstream gene of the anthocyanin metabolism pathway and plays a negative regulatory role in organ and tissue transfer after anthocyanin synthesis (Figure 6). In order to more than understand the molecular mechanism of *plr4* gene in the purple leaf traits of rice, functional validation analysis should be performed by gene overexpression and gene knockout.

Purple leaf traits in rice have attracted extensive attention in terms of both the mechanism of anthocyanin biosynthesis pathway and in the application of hybrid rice breeding. In the breeding process of two-line hybrid rice, regardless of the species or weather, the seeds of two-line sterile line often undergo 3–5% self-hybridization, which leads to large production losses. In our study, the purple leaf traits were controlled by a pair of recessive genes, and were hybridized with green leaf rice after introducing the two-line sterile lines. At the seedling stage and even during the whole growth stage, the hybrid F_1_ plants exhibited green leaves, whereas the plants produced by the self-crossing of the two-line sterile lines showed purple leaves. On the basis of the obvious difference in leaf color, the purple leaf sterile plants could be identified and removed at the seedling stage, thereby ensuring the purity of field hybrids. By backcrossing XianhongB with two-line sterile lines Y58S and GZ63S, we introduced purple leaf traits and bred the purple leaf two-line sterile lines, which were applied to the breeding of two-line hybrid rice.

## 4. Materials and Methods

### 4.1. Materials and Population Construction

The rice variety XianhongB, a fertility restorer line, with purple coloration in the leaves, leaf sheaths, leaf rings, stems, and lemma, was selected as the donor parent. The rice variety Y58S, a male sterile line, with whole green plant and white stigma, was selected as the recipient parent. A set of NILs (BC_3_F_1_) was bred via crossing and back-crossing. In the BC_3_F_2_ to BC_3_F_5_ offspring, population separation and phenotype identification were carried out. We then sequenced the whole genome, and RNA sequencing was conducted for the BC_3_F_5_ and BC_3_F_6_ population, respectively, to map and identify the candidate gene (*plr4*) for the purple leaf traits. The materials were cultivated in an experimental field at the Guangxi Academy of Agricultural Sciences from March to November in 2008–2017 (Figure 1). After flowering, population separation was analyzed, and the data were recorded.

### 4.2. DNA Isolation and Analysis of WGS Data

The healthy leaves of two parents and BC_3_F_5_ individuals were collected at the tillering stage and stored in a −80 °C refrigerator. The genomic DNA was extracted by the modified CTAB (Cetyltrimethyl Ammonium Bromide) method [35]. The purity and integrity of each DNA sample were determined by agarose gel electrophoresis. BSA with next-generation sequencing was conducted on four DNA mixed pools (pl1-pool, pl2-pool, pl3-pool, and pl4-pool) from four extreme phenotypes of pl1, pl2, pl3, and pl4 (Figure 2), and each phenotype contained 40 individuals in the BC_3_F_5_ generation. A total amount of 1.5 μg DNA per sample was used as input material for the DNA sample preparations. Sequencing libraries were generated using Truseq Nano DNA HT Sample preparation kit (Illumina USA), following the manufacturer’s recommendations, and index codes were added to attribute sequences to each sample. The DNA sample was fragmented by sonication to a size of 350 bp, then DNA fragments were end polished, A-tailed, and ligated with the full-length adapter for Illumina sequencing with further PCR amplification. These libraries constructed above were sequenced by an Illumina HiSeq4000 platform and 150 bp paired-end reads were generated with insert size around 350 bp, and sequencing depth of 30× for parental plants and each BC_3_F_5_ generation pool.

Sequencing data were subjected to quality control and mapped to the reference genome. The raw data (raw reads) of fast format was first processed through a series of quality control (QC) procedures in-house C scripts to remove low quality paired reads, which mainly resulted from base-calling duplicates and adapter contamination. The reference genome of *O. sativa* ssp. *japonica* was downloaded from EnsemblGenomes [36] (ftp://ftp.ensemblgenomes.org/pub/plants/release-36/fasta/oryza_sativa/dna/). BWA (Burrows-Wheeler Aligner) was used to align the clean reads of each sample against the reference genome (settings: mem -t 4 -k 32 -M -R) [37]. Alignment files were converted to BAM files using SAMtools software (settings: -bS -t). In addition, potential PCR duplications were removed using SAMtools command “rmdup”. If multiple read pairs have identical external coordinates, only the pair with the highest mapping quality is retained [37]. The Unified Genotyper function was used for variant calling of all sample variants, and s SNPs were selected by using the variant filtration parameters in GATK [38]. The single nucleotide polymorphism index (SNPI) was calculated by using the reading depth information of homozygote single nucleotide polymorphisms in two pools, and the pl1 pool was used as a reference. The SNP index was calculated as the SNP index of the pl1 pool minus the SNP index of the pl2, pl3 and pl4 pool. Whole-genome resequencing was performed by Novogene Bioinformatics Technology Co., Ltd. (Beijing, China).

### 4.3. RNA Extraction and Illumina Sequencing

Total RNA was extracted from the seedling stage to obtain six mixed RNA samples, the leaf sample RNA of the pl3 phenotype homozygous group, and the pl2 phenotype homozygous group. Each group comprised three replicates in the BC_3_F_6_ population (Figure 1 and Figure 2). RNA was extracted and sequenced by Novogene Co. Ltd. (Beijing, China). After the total RNA was extracted from the samples, the RNA was enriched by magnetic beads with Oligo (dT). The obtained RNA to make the fragments into short fragments using divalent cations under elevated temperature in NEBNext^®^ first-strand synthesis reaction buffer (5×), and then the fragmented RNA was used as the template to synthesize the first-strand cDNA using a random hexamer primer and M-MLV reverse transcriptase (RNase H-). Second-strand cDNA synthesis was subsequently performed using DNA polymerase I and RNase H. To select the cDNA fragments with lengths of 250–300 bp, the library fragments were purified with AMPure XP system (Beckman Coulter, Beverly, WV, USA). Following the manufacturer’s protocol, the terminal repair, poly (A) tails were added and sequencing adapter were carried out, and PCR amplification was used to complete the whole library preparation. The constructed library was sequenced on an Illumina Hiseq platform, and 125 bp/150 bp paired-end reads were generated. The reads obtained from the sequencing instruments were filtered to remove the adapters and low-quality reads. High-quality clean reads from all six samples were merged and mapped to the reference sequence.

### 4.4. Quantification of Gene Expression Level and Differential Expression Analysis

The level of gene expression was quantified by FPKM (fragments per kilobase of transcript per million mapped reads) [39]. Differential expression analysis of two conditions/groups (three biological replicates per condition) was performed using the DESeq R package (1.18.0). Genes with an adjusted *p* < 0.05 based on DESeq were considered differentially expressed. The expression level of DEG was higher in purple leaf plants than in green leaf plants, which was considered to be upregulated, whereas those exhibiting the opposite relationship was considered to be downregulated.

### 4.5. GO and KEGG Enrichment Analysis of DEGs

GO enrichment analysis of DEGS was carried out by using GOseq R software package, and gene length bias was corrected. The GO terms with corrected *p* < 0.05 were considered to be significantly rich in DEGS. KEGG is the major public pathway-related database for understanding high-level functions and utilities of the biological system (http://www.genome.jp/kegg/). We used KOBAS software to test the statistical enrichment of DEGs in the KEGG pathways.

### 4.6. qRT-PCR Analysis

Total RNA was extracted from the purple leaf plants and green leaf plants for RNA-Seq, and cDNA was synthesized using the RevertAid™ RT reagent kit (Thermo Fisher Scientific Inc., Waltham, USA). One significant DEG and two DEGs related to anthocyanin exhibiting differential expression patterns were selected for qRT-PCR analysis. The qRT-PCR validation was performed with three techniques repeated. The primers of qRT-PCR were designed by Primer 3 based on the sequence of these genes (Table S9). the QuantiNova™ SYBR^®^ Green PCR kit (Qiagen Inc., Duesseldorf, Germany) was used for the reaction on an ABI 7500 qPCR instrument (Applied Biosystems Inc., Carlsbad, California, USA). Differences in gene expression were calculated using the 2^−ΔΔCt^ method. The *Actin* gene (*LOC4333919*) of rice was used as an internal reference control.

## 5. Conclusions

The *plr4* gene was mapped via genetic analysis, BSA-Seq, RNA-Seq, and qRT-PCR. Three DNA pools were constructed based on the four isolation phenotypes of purple leaf rice. BSA-Seq was used to locate the candidate region of the recessive gene *plr4*, which is associated with the purple leaf of rice. RNA-Seq analysis showed that 10 genes were expressed differently in the candidate region and nearby regions, and only two (*Os04g0577800* and *Os04g0616400*) of them were downregulated, which might be the target recessive genes. qRT-PCR analysis of the three selected genes also confirmed that the expression changes were consistent with the results of RNA-Seq. This study may provide a basis for further understanding the molecular regulation mechanism of purple leaves and provide an effective and powerful scientific basis for the anthocyanin biosynthesis pathway and cross-breeding application in rice.

## Figures and Tables

**Table 1:** The 19 differentially expressed genes demonstrated annotation functions related to the anthocyanin pathway.

Gene_id	Readcount_pl2	Readcount_pl3	Log2foldchange	Pval	Padj	Annotation
*OS02G0207100*	3.083863443	25.98592481	−3.0749	0.00016078	0.031904	UDP-glycosyltransferase
*OS04G0305700*	227.9660245	60.11418403	1.923	2.87 × 10^−^^6^	0.00089272	UDP-glycosyltransferase
*OS04G0525100*	517.0297915	55.58533108	3.2175	7.28 × 10^−^^8^	3.11 × 10^−^^5^	UDP-glycosyltransferase
*OS04G0525200*	192.4804562	24.21323097	2.9908	2.22 × 10^−^^10^	1.78 × 10^−7^	UDP-glycosyltransferase
*OS06G0187500*	3107.154031	953.1108178	1.7049	1.28 × 10^−^^5^	0.0035155	UDP-glycosyltransferase
*OS12G0561900*	11.75742752	50.86821711	−2.1132	0.00024505	0.045572	UDP-glycosyltransferase
*OS01G0735300*	66.19051183	8.444736784	2.9705	0.00020562	0.03935	anthocyanidin 3-O-glucosyltransferase
*OS06G0192100*	3002.334615	101.0286376	4.8932	2.83 × 10^−^^13^	4.05 × 10^−^^10^	anthocyanidin 4-O-glucosyltransferase
*OS07G0503500*	285.5387079	19.21306586	3.8935	2.49 × 10^−^^8^	1.28 × 10^−^^5^	anthocyanidin 5-O-glucosyltransferase
*OS11G0461200*	183.2680426	29.44993347	2.6376	2.74 × 10^−^^5^	0.0069429	anthocyanidin 6-O-glucosyltransferase
*OS04G0557800*	26651.36063	50.70343466	9.0379	9.20 × 10^−^^72^	3.03 × 10^−^^67^	anthocyanin regulatory Lc protein
*OS11G0258700*	37.48606985	0	Inf	6.84 × 10^−^^11^	5.92 × 10^−^^8^	anthocyanin regulatory Lc protein
*OS01G0372500*	8756.71214	16.44143835	9.0569	1.39 × 10^−^^20^	4.15 × 10^−^^17^	Leucoanthocyanidin dioxygenase
*OS01G0633500*	4738.917524	3.00821781	10.621	3.50 × 10^−^^30^	2.88 × 10^−^^26^	Dihydroflavonol−4-reductase
*OS04G0557500*	4723.620711	2.49424188	10.887	1.07 × 10^−^^40^	1.75 × 10^−^^36^	Anthocyanin regulatory R-S protein
*OS04G0630300*	10.54946251	386.0994026	−5.1937	1.24 × 10^−^^9^	8.16 × 10^−^^7^	Anthocyanidin reductase
*OS10G0320100*	938.0204694	98.06073341	3.2579	5.13 × 10^−^^12^	5.63 × 10^−^^9^	Flavonoid 3′-hydroxylase
*OS11G0530600*	814.6837695	15.86922443	5.6819	9.91 × 10^−^^29^	6.52 × 10^−^^25^	Chalcone synthase 1
*OS12G0270900*	383.6491391	8.836561185	5.4402	8.77 × 10^−^^28^	4.81 × 10^−^^24^	Flavonol 3-sulfotransferase

## Supplementary Materials

Supplementary materials can be found at https://www.mdpi.com/1422-0067/20/18/4335/s1.

## Abbreviations

GO	Gene Ontology
KEGG	Kyoto Encyclopedia of Genes and Genomes
qRT-PCR	Quantitative real time-PCR
NILs	Near isogeniclines
BSA-Seq	Bulked segregant analysis with a next-generation sequencing
RNA-Seq	Transcriptome sequencing
FDR	False discovery rate
DEGs	Differentially expressed genes
