# Supplementary material for: Mapping and Identifying a Candidate Gene Plr4, a Recessive Gene Regulating Purple Leaf in Rice, by Using Bulked Segregant and Transcriptome Analysis with Next-Generation Sequencing
Source: Int J Mol Sci. 2019 Sep 4;20(18):4335. doi: 10.3390/ijms20184335 (PMC6769577; doi:10.3390/ijms20184335)
Supplement: Supplementary file 1 [file ijms-20-04335-s001.zip › ijms-577554-supplementary/Figure S2.pdf]

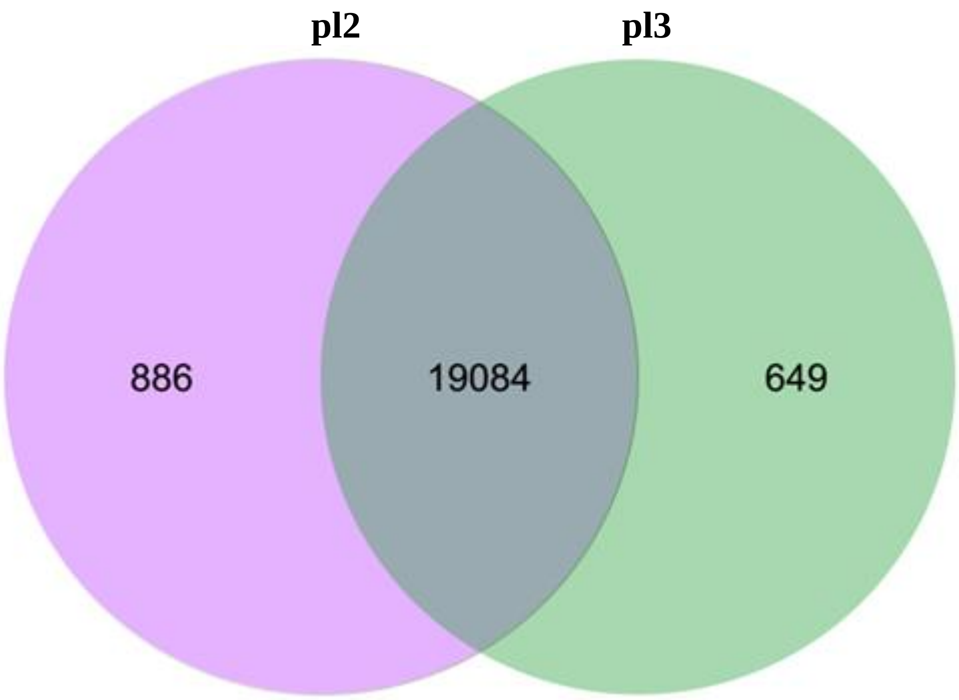

Figure 2. Venn diagram of gene expression. The sum of the numbers in each large circle represents the total number of genes expressed by the group. The overlapping parts of the circle represent the common expression genes among groups. The criterion of gene expression is FPKM > 1.
