# Supplementary material for: Mapping and Identifying a Candidate Gene Plr4, a Recessive Gene Regulating Purple Leaf in Rice, by Using Bulked Segregant and Transcriptome Analysis with Next-Generation Sequencing
Source: Int J Mol Sci. 2019 Sep 4;20(18):4335. doi: 10.3390/ijms20184335 (PMC6769577; doi:10.3390/ijms20184335)
Supplement: Supplementary file 1 [file ijms-20-04335-s001.zip › ijms-577554-supplementary/Figure S3.pdf]

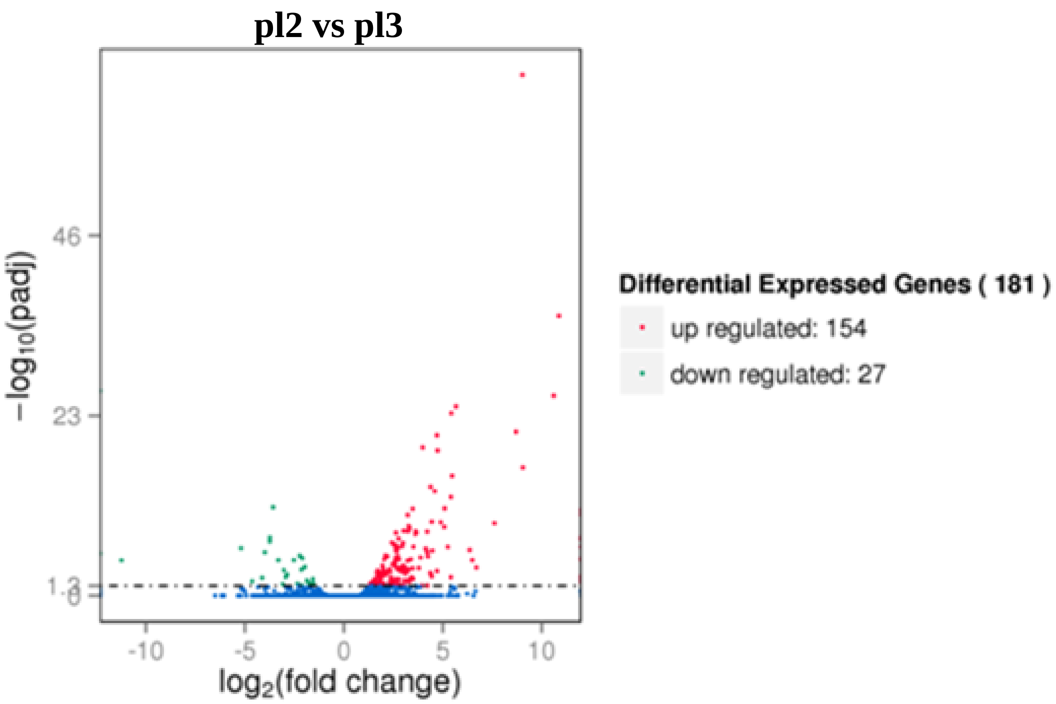


**Figure 3.** Volcanic map of differentially expressed genes. Genes with significant differential expression were indicated by red (up-regulation) and green dots (down-regulation). Genes with no significant differential expression were represented by blue dots; abscissas represent the fold change of genes in different samples; ordinate represents the statistical significance of differences in gene expression changes.
