# Supplementary figures and images for: Mapping and Identifying a Candidate Gene Plr4, a Recessive Gene Regulating Purple Leaf in Rice, by Using Bulked Segregant and Transcriptome Analysis with Next-Generation Sequencing
Source: Int J Mol Sci. 2019 Sep 4;20(18):4335. doi: 10.3390/ijms20184335 (PMC6769577; doi:10.3390/ijms20184335)

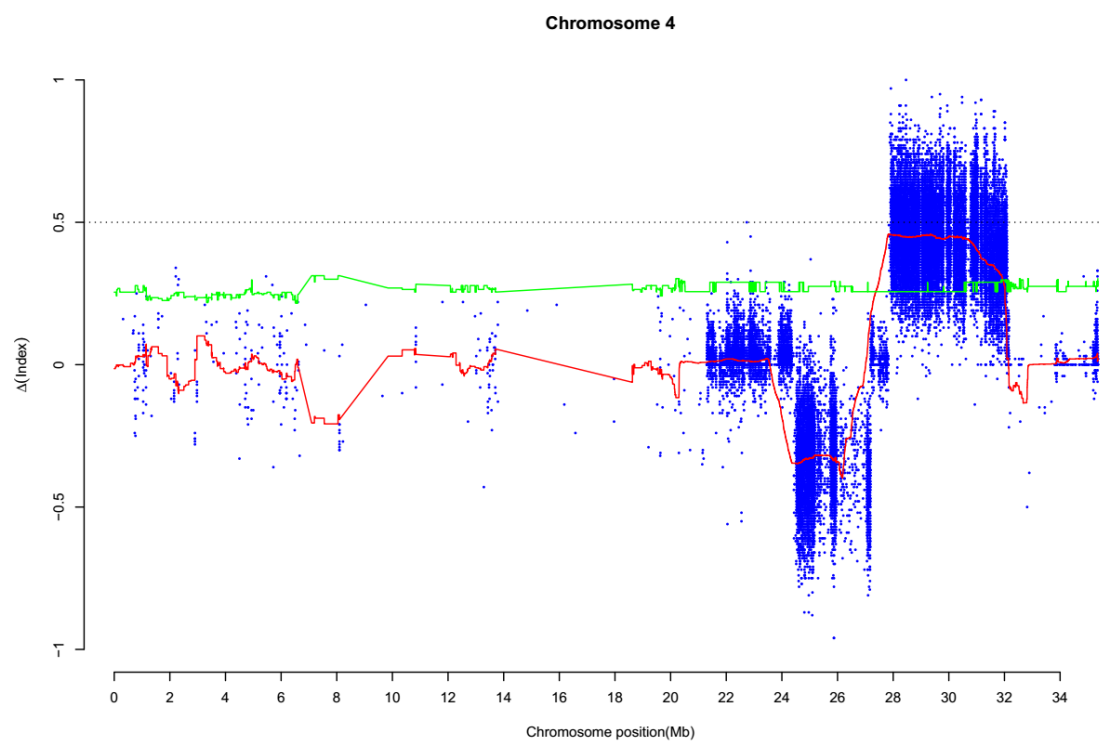

**Figure 1.** Distribution of progeny  $\Delta(\text{SNP-index})$  of p11-pl2 pool in chromosome 4.

Supplement: Supplementary file 1 [file ijms-20-04335-s001.zip › ijms-577554-supplementary/Figure S1.pdf]

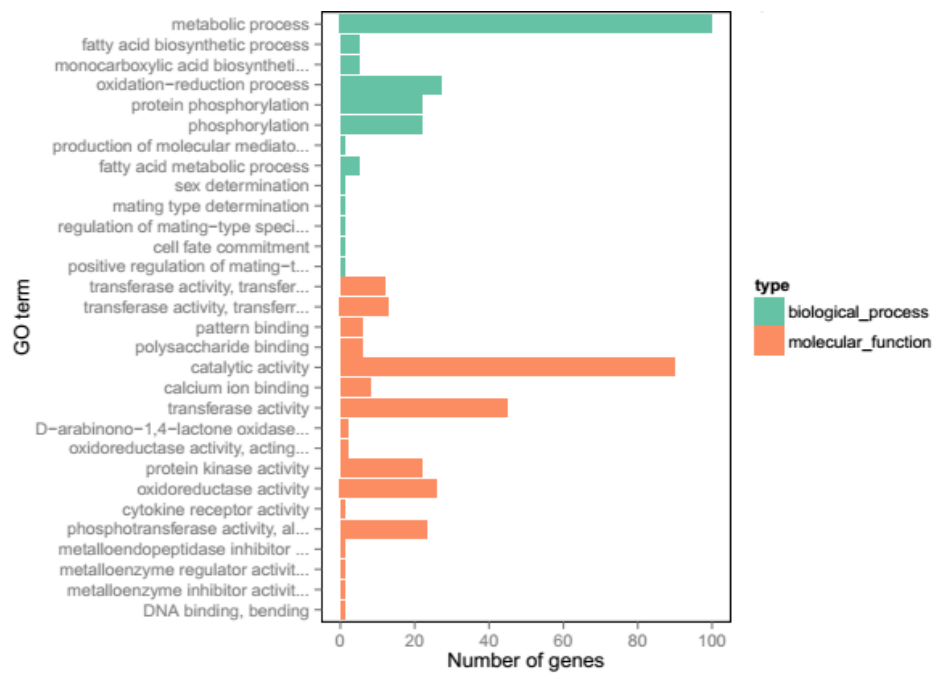

**Figure 4.** Most enriched GO terms.

Supplement: Supplementary file 1 [file ijms-20-04335-s001.zip › ijms-577554-supplementary/Figure S4.pdf]

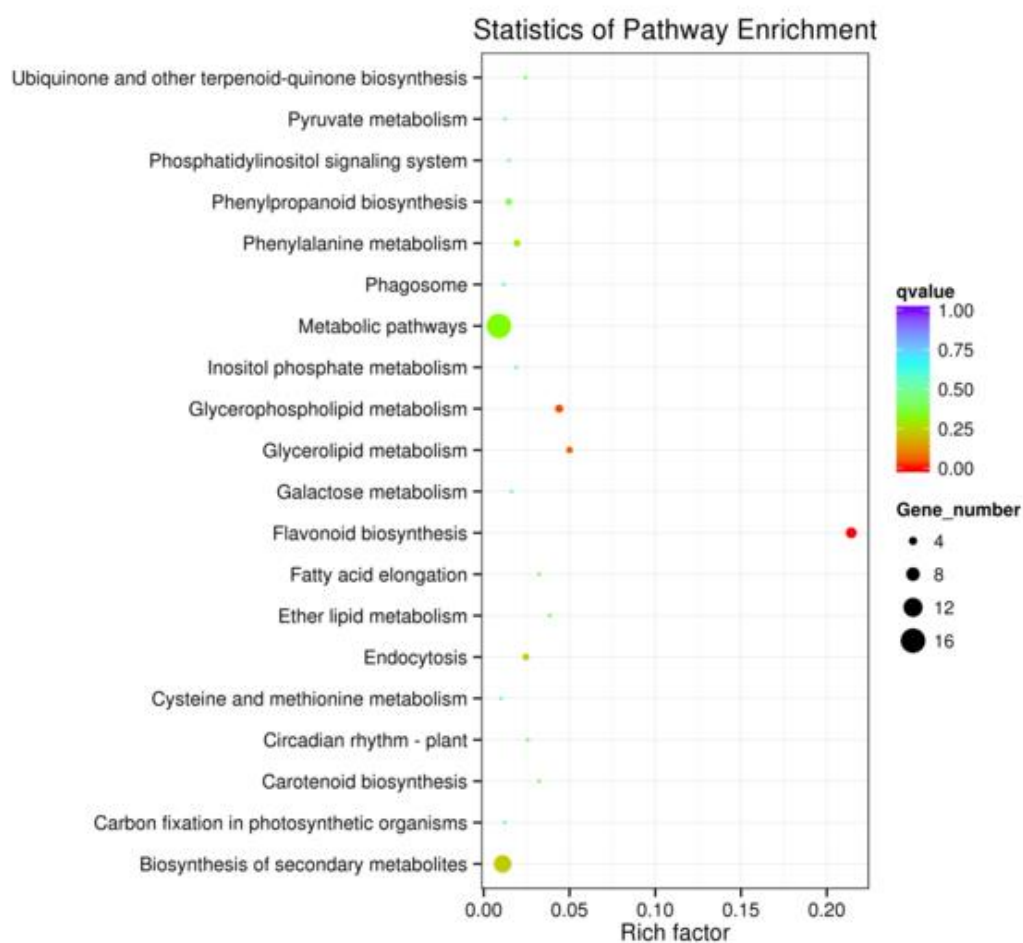

**Figure 5.** Scatter plot of differential gene KEGG enrichment.

Supplement: Supplementary file 1 [file ijms-20-04335-s001.zip › ijms-577554-supplementary/Figure S5.pdf]

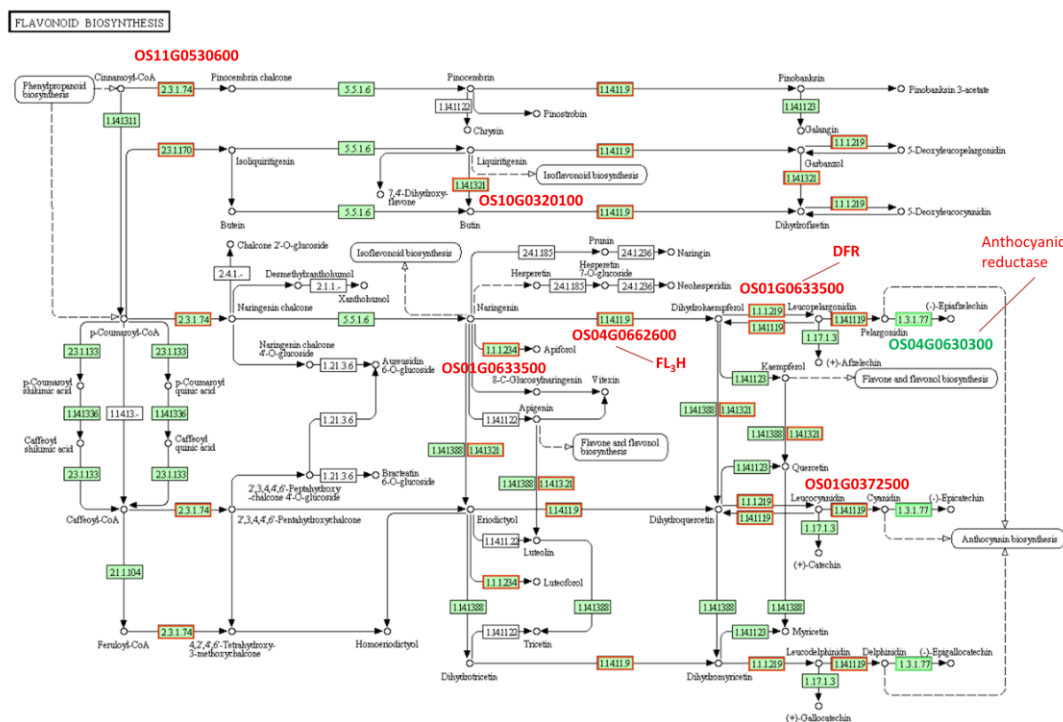

**Figure 6.** Significantly enriched KEGG pathway in the anthocyanin metabolic pathway.

Supplement: Supplementary file 1 [file ijms-20-04335-s001.zip › ijms-577554-supplementary/Figure S6.pdf]
